# Supplementary material for: Content-rich biological network constructed by mining PubMed abstracts
Source: BMC Bioinformatics. 2004 Oct 8;5:147. doi: 10.1186/1471-2105-5-147 (PMC528731; doi:10.1186/1471-2105-5-147)
Supplement: Additional File 2 — The original results of the above study (non-essential files are deleted to keep the file size under the limit set by BMC bioinformatics). [file 1471-2105-5-147-S2.bz2 › chilibotAdditionalFile2/dip50/240ID9063449E787/html/MMP1_TIMP1.html]

 


 **MMP1** and **TIMP1** 
  
Found 2107 abstracts in PubMed, retrieved 50.  
 

 What does Google say? 
 PDF only 
| .edu only 

---

**Interactive relationship** (e.g. stimulation, inhibition, etc)

**Inhibitory relationship**- After injury induced neural degeneration, MMP  [ **MMP1** ]  2 and MT1 MMP  [ **MMP1** ]  levels decreased while TIMP  [ **TIMP1** ]  2 levels increased.  Ref: 12821796 Neuroreport, 2003
- CONCLUSIONS These observations suggest that EPA  [ **TIMP1** ]  attenuates an increase in MMP 1  [ **MMP1** ]  following H R, which may be a basis of protection of myocytes from the adverse effects of H R. p38MAPK phosphorylation may be an important signaling event in this process.  Ref: 12829171 Cardiovasc Res< MedlineTA>Cardiovasc Res, 2003
- We show that TIMP 1  [ **TIMP1** ]  can be transformed into an active inhibitor against MT1 MMP  [ **MMP1** ]  by the mutation of a single residue, namely, threonine 98 to leucine T98L.  Ref: 12869573 J Biol ChemJ Biol Chem, 2003
- The minidegradome constituted of two matrix metalloproteinases MMP  [ **MMP1** ] , i.e. MMP  [ **MMP1** ]  2 and MT1 MMP  [ **MMP1** ] , associated with tissue inhibitor of metalloprotease 2 TIMP  [ **TIMP1** ]  2 and integrin alpha v beta 3 or CD 44, is mainly involved in such invasive program.  Ref: 12888258 Biomed Pharmacother, 2003
- These include increased synthesis of type IV collagen following hyperglycaemia induced alteration of the pattern of podocyte integrin expression, decreased expression of matrix metalloproteinases MMP  [ **MMP1** ]  2 and 3, and increased expression of tissue inhibitor of metalloproteinase TIMP  [ **TIMP1** ] .  Ref: 12845621 J Pathol, 2003
- iii During folic acid 6 weeks treatment, normalization of homocysteine levels was accompanied by a significant reduction in mRNA levels of MMP  [ **MMP1** ]  9 and TIMP 1  [ **TIMP1** ]  in PBMCs, as well as a marked reduction in oxLDL stimulated release of MMP  [ **MMP1** ]  enzyme activity.  Ref: 12814391 Eur J Clin Invest, 2003
**Neutral relationship**- Differing TIMP  [ **TIMP1** ]  orientations in MMP  [ **MMP1** ]  active sites are more likely to result from structural differences in TIMP  [ **TIMP1** ]  AB hairpin loops than from crystal packing artifacts.  Ref: 12834347 Biochemistry, 2003
- Crystal structures of catalytic domains of MMP  [ **MMP1** ]  3 and MT1 MMP  [ **MMP1** ]  bound to TIMP 1  [ **TIMP1** ]  or TIMP  [ **TIMP1** ]  2, respectively, differ in the orientation of the TIMP  [ **TIMP1** ]  in the MMP  [ **MMP1** ]  active site.  Ref: 12834347 Biochemistry, 2003
- Significant negative MMP  [ **MMP1** ]  9, MMP  [ **MMP1** ]  3, MMP  [ **MMP1** ]  8, TIMP  [ **TIMP1** ]  2, total MMP  [ **MMP1** ]  activity and positive MMP  [ **MMP1** ]  2, TIMP 1  [ **TIMP1** ]  correlations were observed with the postoperative day, e.g., total MMP  [ **MMP1** ]  9 day 1, median, 121 range, 12 189 ng ml.  Ref: 12846914 Wound Repair Regen< MedlineTA>Wound Repair Regen, 2003
- Some uncertainty in the N TIMP 1  [ **TIMP1** ]  orientation in the MMP  [ **MMP1** ]  3 active site, coupled with microsecond to millisecond fluctuations of the MMP  [ **MMP1** ]  binding ridge of N TIMP 1  [ **TIMP1** ]  in the complex and flexibility in MMP  [ **MMP1** ]  3 DeltaC S 1 to S 3 subsites, leaves open the possibility that N TIMP 1  [ **TIMP1** ]  might dynamically pivot a few degrees or more in the arc toward the MT1 MMP  [ **MMP1** ]  DeltaC TIMP  [ **TIMP1** ]  2 orientation.  Ref: 12834347 Biochemistry, 2003
- The enzymatic activity of MT1 MMP  [ **MMP1** ]  is regulated by endogenous inhibitors, the tissue inhibitor of metalloproteinases  [ **TIMP1** ]  TIMP  [ **TIMP1** ] .  Ref: 12869573 J Biol ChemJ Biol Chem, 2003
- GLA and eicosapentaenoic acid EPA  [ **TIMP1** ]  caused significant changes in cellular fatty acid composition and increased the percentage of cells with a low MMP  [ **MMP1** ]  after a 96 h exposure period.  Ref: 12838507 Glia< MedlineTA>Glia, 2003

**Non-interactive relationship** (e.g. studied together, co-existance, homology, etc.)

- To study levels and activity of matrix metalloproteinase MMP 1  [ **MMP1** ]  and 9 and their tissue inhibitor TIMP 1  [ **TIMP1** ]  in tears of patients with vernal keratoconjunctivitis VKC, with and without severe corneal damage.  Ref: 12824251 Invest Ophthalmol Vis Sci, 2003
- The expression of matrix metalloproteinase MMP  [ **MMP1** ]  2, MMP  [ **MMP1** ]  9, tissue inhibitor 1 of matrix metalloproteinase TIMP 1  [ **TIMP1** ] , cell adhesion molecule 44 variant 6 CD44v6, HER2 neu and p53 was investigated in 154 patients with head and neck squamous cell carcinoma SCC by ABC and ImmunoMax immunohistochemical method.  Ref: 12861629 J Zhejiang Univ SciJ Zhejiang Univ Sci, 2003
- The ratio of MMP  [ **MMP1** ]  9 to TIMP 1  [ **TIMP1** ]  was significantly increased on admission in both groups and returned to baseline at 48 h.  Ref: 12824721 Cardiology, 2003
- The relationship between MMP  [ **MMP1** ]  9 and its principal inhibitor, tissue inhibitor of metalloproteinase TIMP 1  [ **TIMP1** ]  in voided urine samples was also studied.  Ref: 12855633 Clin Cancer Res< MedlineTA>Clin Cancer Res, 2003
- There were no changes in the expression or activity of MMP  [ **MMP1** ]  inhibitors, TIMP  [ **TIMP1** ] , and 4.  Ref: 12875773 J Am Coll Cardiol, 2003
- The expression of TNFalpha, IL6, MMP 1  [ **MMP1** ] , MMP  [ **MMP1** ]  3, MMP  [ **MMP1** ]  13, TIMP 1  [ **TIMP1** ] , and VEGF was on average higher in ST obtained at arthroscopy.  Ref: 12810425 Ann Rheum Dis< MedlineTA>Ann Rheum Dis, 2003
- Among the patients with strong grades 2 and 3 MMP  [ **MMP1** ]  9 staining positivity for TIMP 1  [ **TIMP1** ]  indicated a trend toward a more favorable prognosis.  Ref: 12890147 Eur J Haematol, 2003
- The results show a correlation between MMP  [ **MMP1** ]  and TIMP  [ **TIMP1** ]  levels and the stages of neural degeneration, regeneration and recovery of the olfactory epithelium following injury.  Ref: 12821796 Neuroreport, 2003
- , TIMP 1  [ **TIMP1** ]  and TIMP  [ **TIMP1** ]  2 in synovial fluid from patients n = 56 with different degrees of either chondral lesions or knee arthritis identified and classified by arthroscopy.Zymographies, Western blotting and ELISA tests have been used to correlate the disease stage, as determined by arthroscopy, and both the amount and the activation state of different MMPs and of their inhibitors.Analysis of data obtained demonstrates that the degree of cartilage degradation, as seen by arthroscopy, is strictly related to the activity of some synovial MMPs, in particular MMP  [ **MMP1** ]  2 and MMP  [ **MMP1** ]  13 and on reduced inhibitory effect of MMP  [ **MMP1** ]  2 by TIMP  [ **TIMP1** ]  2.  Ref: 12810159 Clin Biochem, 2003
- Despite similar cellular inflammatory patterns both in COPD and IPF sputa, marked differences were observed with regard to MMP  [ **MMP1** ]  9 TIMP 1  [ **TIMP1** ]  balance.  Ref: 12814147 Respir Med, 2003
- This study was designed to examine the effect of eicosapentanoic acid EPA  [ **TIMP1** ] , one of the major components in fish oil, on the modulation of MMP 1  [ **MMP1** ]  expression in response to H R in cultured adult rat myocytes.  Ref: 12829171 Cardiovasc Res< MedlineTA>Cardiovasc Res, 2003
- Nonetheless, when divided into tertiles, MMP  [ **MMP1** ]  3 and TIMP 1  [ **TIMP1** ]  levels in the highest tertile were positively associated with the presence of carotid artery lesions odds ratios=3.4 and 2.0, confidence intervals 1.7 13.9 and 1.3 7.9, respectively.  Ref: 12860260 Atherosclerosis, 2003
- In contrast, cell associated protein levels of TIMP 1  [ **TIMP1** ] , an inhibitor of MMP  [ **MMP1** ]  9, are insensitive to the shear regimen.  Ref: 12816956 J Biol Chem< MedlineTA>J Biol Chem, 2003
- Gelatinase activities and immunohistochemical localization of MMP  [ **MMP1** ] ,, and 13 and TIMP 1  [ **TIMP1** ]  and 2 were examined in mandibular condyle cartilage of neonatal mice up to 18 months old.  Ref: 12827373 Histochem Cell Biol< MedlineTA>Histochem Cell Biol, 2003
- Global Orientation of Bound MMP  [ **MMP1** ]  3 and N TIMP 1  [ **TIMP1** ]  in Solution via Residual Dipolar Couplings,.  Ref: 12834347 Biochemistry, 2003
- Gene expressions of type I and II collagen, metalloproteinase 1 and 3 MMP 1  [ **MMP1** ]  and 3, and tissue inhibitor of metalloproteinase 1  [ **TIMP1** ]  TIMP 1  [ **TIMP1** ]  as well as the IL 1beta induced gene expressions of MMP 1  [ **MMP1** ]  and 3 were analyzed by reverse transcription polymerase chain reaction RT PCR.  Ref: 12880581 Osteoarthritis CartilageOsteoarthritis Cartilage, 2003
- The purpose of this study was to characterize effects of cytokines, a phorbol ester, and prostanoids on the expression of MMP  [ **MMP1** ] , 3, and 9 and tissue inhibitors of metalloproteinases TIMP 1  [ **TIMP1** ]  and 2 in cultured human TM cells.  Ref: 12882798 Invest Ophthalmol Vis Sci, 2003
- Effects of a vegetable extract from Lupinus albus LU105 on the production of matrix metalloproteinases  **MMP1** , MMP2, MMP9 and tissue inhibitor of metalloproteinases  [ **TIMP1** ]   **TIMP1** , TIMP2 by human gingival fibroblasts in culture.  Ref: 12802622 Clin Oral Investig< MedlineTA>Clin Oral Investig, 2003
- Both, TIMP  [ **TIMP1** ]  2 and MMP  [ **MMP1** ]  2 were increased in the RV only after 14 days p <.  Ref: 12841632 Mol Cell Biochem, 2003
- We determined serum levels of MMP  [ **MMP1** ]  3, MMP  [ **MMP1** ]  9, TIMP 1  [ **TIMP1** ]  and TIMP  [ **TIMP1** ]  2 in dyslipidemic subjects and compared them to those of age and sex matched normolipidemic healthy controls.  Ref: 12860260 Atherosclerosis, 2003
- RT was followed by a sharp decrease in plasma MMP  [ **MMP1** ]  9 within the first 2 weeks, but without a corresponding change in TIMP 1  [ **TIMP1** ] .  Ref: 12829155 Int J Radiat Oncol Biol Phys< MedlineTA>Int J Radiat Oncol Biol Phys, 2003
- Serum levels of IL 6, interstitial collagenase  [ **MMP1** ]  MMP 1  [ **MMP1** ] , stromelysin 1 MMP  [ **MMP1** ]  3, gelatinase B MMP  [ **MMP1** ]  9, TIMP 1  [ **TIMP1** ]  and TIMP  [ **TIMP1** ]  2 were assessed with an ELISA technique in 30 RA patients.  Ref: 12879774 Pol Arch Med Wewn, 2003
- Thus LU105 seems to offer an opportunity to restore a correct balance between MMP2, MMP9,  **MMP1** , and their natural inhibitors, i.e.,  **TIMP1**  and TIMP2 in human inflamed gingiva.  Ref: 12802622 Clin Oral Investig< MedlineTA>Clin Oral Investig, 2003
- Thus, serum levels of MMP  [ **MMP1** ]  and TIMP 1  [ **TIMP1** ]  are significantly elevated in asymptomatic hyperlipidemic subjects at high cardiovascular risk.  Ref: 12860260 Atherosclerosis, 2003
- ... MMP  [ **MMP1** ]  8 and 14 and tissue inhibitor of metalloproteinase TIMP 1  [ **TIMP1** ]  were not altered, in corneal epithelial cells.  Ref: 12824233 Invest Ophthalmol Vis Sci, 2003
- Our results showed the following i Compared with healthy control subjects n = 9, patients with hyperhomocysteinaemia n = 9 had elevated mRNA levels of MMP  [ **MMP1** ]  9 and tissue inhibitors of metalloproteinases 1 TIMP 1  [ **TIMP1** ]  in freshly isolated peripheral blood mononuclear cells PBMCs, which were positively correlated with homocysteine and negatively correlated with folate and vitamin B12 levels.  Ref: 12814391 Eur J Clin Invest, 2003
- Both  **TIMP1**  and  **MMP1**  showed positive correlation with each other and with previously determined levels of mRNA for both interleukin 1beta IL 1beta and IL 6 in this patient series when considering all patients individually, but neither correlated with tumor necrosis factor alpha.Patients with deteriorating heart failure have increased expression of  **TIMP1**  and  **MMP1**  mRNA.  Ref: 12873541 J Heart Lung Transplant, 2003
- MMP  [ **MMP1** ]  2, MMP  [ **MMP1** ]  9, MMP  [ **MMP1** ]  8, TIMP 1  [ **TIMP1** ]  and TIMP  [ **TIMP1** ]  2 were detected by immunohistochemistry and the Western blot technique.  Ref: 12873454 Exp Eye Res, 2003
- Organ cultures were performed using explants from the skin of three patients from the same family with recessive dystrophic epidermolysis bullosa to reveal and quantify the expression of MMP 1  [ **MMP1** ]  collagenase 1, MMP  [ **MMP1** ]  2 and MMP  [ **MMP1** ]  9 gelatinases A and B, MMP  [ **MMP1** ]  3 stromelysin 1, TIMP 1  [ **TIMP1** ] , and TIMP  [ **TIMP1** ]  2, and to compare the results with those obtained with two human control skins, with the same experimental conditions.  Ref: 12880418 J Invest Dermatol, 2003
- Our findings also provide a potential mechanistic explanation for MMP  [ **MMP1** ]  TIMP  [ **TIMP1** ]  selectivity.  Ref: 12869573 J Biol ChemJ Biol Chem, 2003
- OBJECTIVES The present study was carried out to clarify the role of matrix metalloproteinase 2 and 9 MMP  [ **MMP1** ]  2 and MMP  [ **MMP1** ]  9, tissue inhibitor of metalloproteinase 1  [ **TIMP1** ]  TIMP 1  [ **TIMP1** ]  and the extent of neovascularization in the clinicopathologic behavior of non Hodgkin s lymphomas.  Ref: 12890147 Eur J Haematol, 2003
- Fitting of the RDCs to the X ray structures of the complexes suggests general agreement with the orientation of crystalline MMP  [ **MMP1** ]  3 DeltaC and TIMP 1  [ **TIMP1** ]  and a large disparity from the orientation of crystalline MT1 MMP  [ **MMP1** ]  DeltaC and TIMP  [ **TIMP1** ]  2.  Ref: 12834347 Biochemistry, 2003
- We hypothesized that matrix metalloproteinases MMPs and their endogenous inhibitors, the tissue inhibitors of MMPs TIMPs, would be implicated in the mechanisms that underlie deteriorating heart failure.Gridded macro array filters were used to provide a broad overview of MMP  [ **MMP1** ]  and TIMP  [ **TIMP1** ]  mRNA expression in heart failure.  Ref: 12873541 J Heart Lung Transplant, 2003
- Platelet derived growth factor BB was generally ineffective in modulating MMP  [ **MMP1** ]  and TIMP  [ **TIMP1** ]  levels.  Ref: 12882798 Invest Ophthalmol Vis Sci, 2003
- So we suspected that the phenotypic variability for the same mutation could be linked to other genetic or environmental factors, as a particular balance between MMP  [ **MMP1** ]  and TIMP  [ **TIMP1** ] .  Ref: 12880418 J Invest Dermatol, 2003
- Our results showed a significant decrease of collagenases MMP  [ **MMP1** ]  8 and MMP  [ **MMP1** ]  13 with an increase of TIMP 1  [ **TIMP1** ]  and transforming growth factor beta.  Ref: 12882763 Am J Physiol Lung Cell Mol PhysiolAm J Physiol Lung Cell Mol Physiol, 2003
- We have compared sputum concentrations of MMP  [ **MMP1** ]  9, TIMP 1  [ **TIMP1** ]  and the controlling cytokine tumor necrosis factor TNF alpha in patients with COPD, IPF and healthy subjects.  Ref: 12814147 Respir Med, 2003
- In the present study, we investigated the effects of decorin on the production of metalloproteinases MMP  [ **MMP1** ] , and 13, tissue inhibitors of metalloproteinases TIMP  [ **TIMP1** ]  and cytokines TGF beta, IL 1beta, IL 4 and TNF alpha.  Ref: 12853035 Matrix Biol, 2003
- Increased expression of extracellular matrix regulators  **TIMP1**  and  **MMP1**  in deteriorating heart failure.  Ref: 12873541 J Heart Lung Transplant, 2003
- However, we were unable to demonstrate any differences in the constitutive productions and secretions of MMP  [ **MMP1** ]  2, MT1 MMP  [ **MMP1** ] , and TIMP  [ **TIMP1** ]  2, among the mock treated, Ad lacZ transduced, and Ad p16 transduced cells.  Ref: 12883667 Oncol RepOncol Rep, 2003
- Concentrations of MMP  [ **MMP1** ]  9 and the MMP  [ **MMP1** ]  9 TIMP 1  [ **TIMP1** ]  ratio were increased in COPD P< ; 0.001 vs. IPF and controls.  Ref: 12814147 Respir Med, 2003
- Plasma concentrations of MMP  [ **MMP1** ]  2, MMP  [ **MMP1** ]  9 and tissue inhibitor of metalloproteinase TIMP 1  [ **TIMP1** ]  were determined on admission, and after 24 h, 48 h, 1 week, 4 weeks, 3 months and 6 months.  Ref: 12824721 Cardiology, 2003
- Pretreatment of myocytes with EPA  [ **TIMP1** ] , but not AA, resulted in a reduction in LDH release, and attenuation of p38MAPK phosphorylation and MMP 1  [ **MMP1** ]  activity and expression in response to H R all P< ; 0.05 vs. H R alone.  Ref: 12829171 Cardiovasc Res< MedlineTA>Cardiovasc Res, 2003
- So we can suspect that recessive dystrophic epidermolysis bullosa phenotypic variability could be related to patients collagenase activity heterogeneity, linked to imbalance between MMP 1  [ **MMP1** ]  and TIMP 1  [ **TIMP1** ] .  Ref: 12880418 J Invest Dermatol, 2003
- However, following 35 days of neurogenesis and cell replacement TIMP  [ **TIMP1** ]  2 and MT1 MMP  [ **MMP1** ]  returned to control levels.  Ref: 12821796 Neuroreport, 2003
- Collagenase and stromelysin activities have been found to be overexpressed in skin cultures of some recessive dystrophic epidermolysis bullosa patients, and tissue destruction in the disease process might result from an imbalance of metalloproteinases MMP  [ **MMP1** ]  over tissueinhibitor of metalloproteinases TIMP  [ **TIMP1** ] .  Ref: 12880418 J Invest Dermatol, 2003
- In this study we examined the sequential expression of several matrix metalloproteinases MMP  [ **MMP1** ] , tissue inhibitors of metalloproteinases TIMP  [ **TIMP1** ] , and growth factors as well as the presence of apoptosis in a model of pulmonary fibrosis induced in rats with paraquat and hyperoxia.  Ref: 12882763 Am J Physiol Lung Cell Mol PhysiolAm J Physiol Lung Cell Mol Physiol, 2003
- Here, we identify the surface epitopes that render TIMP 1  [ **TIMP1** ]  inactive against MT1 MMP  [ **MMP1** ] .  Ref: 12869573 J Biol ChemJ Biol Chem, 2003
- RESULTS Western blot analysis demonstrated the presence of MMP 1  [ **MMP1** ] , MMP  [ **MMP1** ]  2, MMP  [ **MMP1** ]  9, and MMP  [ **MMP1** ]  12, as well as small quantities of tissue inhibitor of metalloproteinases  [ **TIMP1** ]  TIMP 1  [ **TIMP1** ]  and TIMP  [ **TIMP1** ]  2 in protein isolates from normal and varicose veins.  Ref: 12844106 J Vasc Surg, 2003
- Low dose estrogen could decrease the ratio of MMP 1  [ **MMP1** ]  TIMP 1  [ **TIMP1** ]  and MMP  [ **MMP1** ]  3 TIMP 1  [ **TIMP1** ] , which could even be decreased by progestin.  Ref: 12887766 Zhonghua Yi Xue Za Zhi, 2003
- Linear regression and correlation analysis revealed that there was close positive relationship P< ; 0.05 between the expression of MMP  [ **MMP1** ]  2 and MMP  [ **MMP1** ]  9, TIMP 1  [ **TIMP1** ]  and CD44v6, HER2 neu and MMP  [ **MMP1** ]  9, MMP  [ **MMP1** ]  2 and p53.  Ref: 12861629 J Zhejiang Univ SciJ Zhejiang Univ Sci, 2003
- **TIMP1**  and  **MMP1**  significantly decreased when LU105 was added in the culture media of gingival fibroblasts derived from inflamed tissue compared with control fibroblasts.  Ref: 12802622 Clin Oral Investig< MedlineTA>Clin Oral Investig, 2003
- Our findings establish an unexpected, MMP  [ **MMP1** ]  independent mechanism for TIMP  [ **TIMP1** ]  2 inhibition of endothelial cell proliferation in vitro and reveal an important component of the antiangiogenic effect of TIMP2 in vivo.  Ref: 12887919 Cell, 2003
- , MMP  [ **MMP1** ]  and 9 activities were upregulated, and TIMP  [ **TIMP1** ]  2 was expressed.  Ref: 12873454 Exp Eye Res, 2003
- Lung and breast cancer resulted in very high plasma levels of MMP  [ **MMP1** ]  9 126 893 ng mL and TIMP 1  [ **TIMP1** ]  496 85 ng mL in all subjects studied before initiation of RT.  Ref: 12829155 Int J Radiat Oncol Biol Phys< MedlineTA>Int J Radiat Oncol Biol Phys, 2003
- The expression of MMP  [ **MMP1** ]  2, MMP  [ **MMP1** ]  9, TIMP 1  [ **TIMP1** ] , CD44v6, HER2 neu and p53 was found in cancer cells in 87.01%, 85.71%, 68.18%, 98.05%, 55.19% and 50.65% cases respectively.  Ref: 12861629 J Zhejiang Univ SciJ Zhejiang Univ Sci, 2003
- The orientation in solution of N TIMP 1  [ **TIMP1** ]  in the MMP  [ **MMP1** ]  3 active site has been investigated using residual dipolar couplings RDCs.  Ref: 12834347 Biochemistry, 2003
- RESULTS Baseline plasma YKL 40, MMP  [ **MMP1** ]  2, PIIINP and TIMP 1  [ **TIMP1** ]  were significantly increased in patients compared to normal controls.  Ref: 12825876 Scand J Gastroenterol, 2003
- This compares with plasma MMP  [ **MMP1** ]  9 and TIMP 1  [ **TIMP1** ]  values in healthy volunteers of 29 11 ng mL and 436 86 ng mL, respectively.  Ref: 12829155 Int J Radiat Oncol Biol Phys< MedlineTA>Int J Radiat Oncol Biol Phys, 2003
- Among the 53 SF samples 9 RA, 24 GA, and 20 OA analyzed for MMP  [ **MMP1** ]  9 and tissue inhibitor of metalloproteinase TIMP 1  [ **TIMP1** ]  antigen levels by ELISA, MMP  [ **MMP1** ]  9 antigen levels were elevated tenfold in GA SF compared with OA SF.  Ref: 12858344 J Cell Biochem, 2003
- Serum levels of MMP  [ **MMP1** ]  3, MMP  [ **MMP1** ]  9 and TIMP 1  [ **TIMP1** ]  were significantly increased in hyperlipidemic subjects versus controls 5 9 and 15%, respectively.  Ref: 12860260 Atherosclerosis, 2003
- Low MMP  [ **MMP1** ]  9 TIMP 1  [ **TIMP1** ]  ratios in patients with nonmuscle invasive tumors were associated with higher recurrence rates P = 0.0035.  Ref: 12855633 Clin Cancer Res< MedlineTA>Clin Cancer Res, 2003
- Rigid body docking of MMP  [ **MMP1** ]  3 and N TIMP 1  [ **TIMP1** ]  X ray coordinates using RDCs and intermolecular NOEs provided a time averaged orientation in solution differing from the crystal structure by a 5 degrees rotation toward the MT1 MMP  [ **MMP1** ]  DeltaC TIMP  [ **TIMP1** ]  2 orientation.  Ref: 12834347 Biochemistry, 2003
- Extracellular matrix molecule degradation related genes, including matrix metalloproteinase MMP  [ **MMP1** ]  10, MMP  [ **MMP1** ]  13, MMP  [ **MMP1** ]  15, tissue inhibitor of metalloproteinase TIMP  [ **TIMP1** ]  2, TIMP  [ **TIMP1** ]  3, plasminogen and plasminogen activator, were also highly expressed in the pre eclamptic placenta, compared to the normal placenta.  Ref: 12866918 Br J Biomed Sci, 2003
- Finally, we observed a decrease of MMP 1  [ **MMP1** ] , MMP  [ **MMP1** ]  3, MMP  [ **MMP1** ]  9, and MT1 MMP  [ **MMP1** ] , both at the mRNA and at the protein levels, in E cadherin positive clones whereas no changes in MMP  [ **MMP1** ]  2, TIMP 1  [ **TIMP1** ] , or TIMP  [ **TIMP1** ]  2 were observed when compared with control clones.  Ref: 12875984 Am J Pathol, 2003
- Precise mRNA levels of  **TIMP1** ,  **MMP1** , and beta spectrin were determined using quantitative real time reverse transcription polymerase chain reaction RT PCR of myocardial samples from 27 patients with deteriorating heart failure who underwent LVAD implantation, from 17 patients with stable ESF who underwent elective heart transplantation, and from 28 donor organs with good hemodynamic function.Gridded macro arrays analysis of pooled failing heart samples determined that  **TIMP1**  mRNA was the most readily detectable TIMP  [ **TIMP1** ]  in failing myocardium.  Ref: 12873541 J Heart Lung Transplant, 2003
- Additionally, compared to the control conjunctival fibroblasts, an increased expression of TGF beta1 was detected in fibroblasts isolated from the conjunctival tissues of patient with EBA.This study suggests that there is up regulation in the production of collagens type I and III, collagen binding protein HSP47, matrix degrading collagenases MMP 1  [ **MMP1** ]  and 14, and their inhibitory enzymes TIMP  [ **TIMP1** ]  and 3 during the process of conjunctival matrix remodeling in the patient with EBA.  Ref: 12824005 Cytokine, 2003
- These high baseline plasma levels of MMP  [ **MMP1** ]  9 were reduced in the first 2 weeks of RT in 7 of 8 subjects, and TIMP 1  [ **TIMP1** ]  plasma levels remained high in all subjects.  Ref: 12829155 Int J Radiat Oncol Biol Phys< MedlineTA>Int J Radiat Oncol Biol Phys, 2003
- The majority of these processes requires a balanced equilibrium between the activity of matrix metalloproteinases MMP  [ **MMP1** ]  and their natural tissue inhibitors TIMP  [ **TIMP1** ] .  Ref: 12879657 Ceska Gynekol, 2003
- The protein synthesis of MMP 1  [ **MMP1** ]  and 3 and TIMP 1  [ **TIMP1** ]  was determined by Western blotting.RESULTS The treatment of chondrocytes with DHEA did not affect chondrocyte proliferation or GAG synthesis up to 100μM of concentration.  Ref: 12880581 Osteoarthritis CartilageOsteoarthritis Cartilage, 2003
- Immunohistological analysis was performed to detect T cells, plasma cells, macrophages, fibroblast like synoviocytes FLS, and the expression of interleukin IL 1beta, IL6, tumour necrosis factor alpha TNFalpha, MMP 1  [ **MMP1** ] , MMP  [ **MMP1** ]  3, MMP  [ **MMP1** ]  13, TIMP 1  [ **TIMP1** ] , and VEGF.  Ref: 12810425 Ann Rheum Dis< MedlineTA>Ann Rheum Dis, 2003
- We suggest that assessing the serum IL 6, MMP 1  [ **MMP1** ] , MMP  [ **MMP1** ]  3, MMP  [ **MMP1** ]  9 and TIMP 1  [ **TIMP1** ]  levels may be helpful in the prediction of the RA activity.  Ref: 12879774 Pol Arch Med Wewn, 2003
- Increased levels and activity of MMP 1  [ **MMP1** ]  and 9 and an imbalance between MMPs and TIMP  [ **TIMP1** ]  may be involved in the pathogenesis of VKC.  Ref: 12824251 Invest Ophthalmol Vis Sci, 2003
- Unveiling the surface epitopes that render tissue inhibitor of metalloproteinase TIMP 1  [ **TIMP1** ]  inactive against membrane type 1 matrix metalloproteinase MT1 MMP  [ **MMP1** ] .  Ref: 12869573 J Biol ChemJ Biol Chem, 2003
- The invasive potential of Ad p16 transduced cells was evaluated using a Matrigel invasion assay, and trimolecular complex MMP  [ **MMP1** ]  2 MT1 MMP  [ **MMP1** ]  TIMP  [ **TIMP1** ]  2 synthesis was proven by zymography and Western blotting.  Ref: 12883667 Oncol RepOncol Rep, 2003
- Whilst TIMP  [ **TIMP1** ] , and 4 are potent inhibitors against MT1 MMP  [ **MMP1** ] , TIMP 1  [ **TIMP1** ]  displays negligible inhibitory activity against the enzyme.  Ref: 12869573 J Biol ChemJ Biol Chem, 2003
- Matrix metalloproteinases MMP  [ **MMP1** ]  2 and 9 and tissue inhibitors of matrix metalloproteinases TIMP 1  [ **TIMP1** ]  and 2 during the course of experimental necrotizing herpetic keratitis.  Ref: 12873454 Exp Eye Res, 2003
- Decorin infection resulted in decreased protein levels of MMP 1  [ **MMP1** ]  and MMP  [ **MMP1** ]  3 whereas MMP  [ **MMP1** ]  2 and TIMP  [ **TIMP1** ]  2 secretion was increased.  Ref: 12853035 Matrix Biol, 2003
- In contrast, plasma MMP  [ **MMP1** ]  3 levels, which are generally increased with inflammation, were elevated in only 1 of 5 subjects.Lung and breast cancer are associated with high plasma levels of MMP  [ **MMP1** ]  9 and TIMP 1  [ **TIMP1** ] .  Ref: 12829155 Int J Radiat Oncol Biol Phys< MedlineTA>Int J Radiat Oncol Biol Phys, 2003
- We demonstrate here that TIMP  [ **TIMP1** ]  2 abrogates angiogenic factor induced endothelial cell proliferation in vitro and angiogenesis in vivo independent of MMP  [ **MMP1** ]  inhibition.  Ref: 12887919 Cell, 2003
- Three laboratory techniques were performed enzyme linked immunosorbent assays MMP 1  [ **MMP1** ] , MMP  [ **MMP1** ]  3, TIMP 1  [ **TIMP1** ] , TIMP  [ **TIMP1** ]  2, gelatinase activity assays MMP  [ **MMP1** ]  2, MMP  [ **MMP1** ]  9, and quenched fluorescent substrate hydrolysis total MMP  [ **MMP1** ]  activity.  Ref: 12846914 Wound Repair Regen< MedlineTA>Wound Repair Regen, 2003
- We used Western blot and immunohistochemistry to determine whether MMP  [ **MMP1** ]  2 and associated proteins TIMP  [ **TIMP1** ]  2 and membrane type 1 matrix metalloproteinase MT1 MMP  [ **MMP1** ]  are present in the olfactory epithelium of mice.  Ref: 12821796 Neuroreport, 2003
- A low urinary MMP  [ **MMP1** ]  9 TIMP 1  [ **TIMP1** ]  ratio may indicate a higher risk of intraluminal nonmuscle invasive tumor recurrence and may assist in planning follow up surveillance protocols.  Ref: 12855633 Clin Cancer Res< MedlineTA>Clin Cancer Res, 2003
- Reverse transcription polymerase chain reaction were used to detect the mRNA expression of MMP 1  [ **MMP1** ] , MMP  [ **MMP1** ]  3, TIMP 1  [ **TIMP1** ]  and IL 1beta in the OA synovia to determine the effect of estrogen and progestin.  Ref: 12887766 Zhonghua Yi Xue Za Zhi, 2003
- MMP  [ **MMP1** ]  9, MMP  [ **MMP1** ]  13 and TIMP 1  [ **TIMP1** ]  were not affected by decorin infection.  Ref: 12853035 Matrix Biol, 2003
- 0.001, and the MMP  [ **MMP1** ]  inhibitor TIMP  [ **TIMP1** ]  2 P = 0.025.  Ref: 12893184 Gynecol Oncol, 2003
- OBJECTIVE To observe the effect of different dose of estrogen or estrogen plus progestin on the mRNA expression of MMP 1  [ **MMP1** ] , MMP  [ **MMP1** ]  3, TIMP 1  [ **TIMP1** ]  and IL 1beta in synovia of female New Zealand White rabbit OA model.  Ref: 12887766 Zhonghua Yi Xue Za Zhi, 2003
- BACKGROUND The possible markers of liver fibrosis plasma YKL 40, PIIINP, MMP  [ **MMP1** ]  2 and TIMP 1  [ **TIMP1** ]  were measured at the start t0 and end of treatment t12 with alpha interferon and ribavirin and repeated at 6 months follow up t18 in 51 patients with chronic hepatitis C.  Ref: 12825876 Scand J Gastroenterol, 2003
- The expression of collagenases MMP  [ **MMP1** ]  and 13 and tissue inhibitor of metalloproteinases  [ **TIMP1** ]  TIMP 1  [ **TIMP1** ]  was altered in OA cartilage, but no difference was detected between lesion and distal sites in the same jointi.e. no correlation was found between senescent cells and proteinase inhibitor expression.  Ref: 12882354 Aging Cell, 2002
- Furthermore for this patient we evidenced higher amounts of MMP 1  [ **MMP1** ]  and also a lower TIMP 1  [ **TIMP1** ]  amount in his unlesioned and lesioned skin compared with the other two affected patients and with healthy control donors.  Ref: 12880418 J Invest Dermatol, 2003
- Sandwich enzyme linked immunoassays ELISA were used to measure plasma MMP  [ **MMP1** ]  3, MMP  [ **MMP1** ]  9, and TIMP 1  [ **TIMP1** ]  levels.  Ref: 12829155 Int J Radiat Oncol Biol Phys< MedlineTA>Int J Radiat Oncol Biol Phys, 2003
- However, the mRNAs coding for urokinase type PA uPA, membrane type 1 MMP  [ **MMP1** ]  MT1 MMP  [ **MMP1** ] , and tissue inhibitor of metalloproteinases  [ **TIMP1** ]  type 3 TIMP  [ **TIMP1** ]  3 were constantly expressed in the mouse CL throughout its whole life span.  Ref: 12890575 Mol Cell Endocrinol, 2003
- METHODS Paraffin embedded histologic sections from 57 patients with aggressive non Hodgkin s lymphomas were stained with MMP  [ **MMP1** ]  2, MMP  [ **MMP1** ]  9, TIMP 1  [ **TIMP1** ] , and factor VIII antibodies to correlate the expression of these markers to the clinical disease characteristics.  Ref: 12890147 Eur J Haematol, 2003
- Does the release of plasma matrix metalloproteinase 9 MMP  [ **MMP1** ]  9 by radiation activated airway epithelial cells and infiltrating inflammatory cells play a role in the radiation damage or repair process in the lungs ? We evaluated lung damage by ionizing radiation using plasma levels of MMP  [ **MMP1** ]  9, tissue inhibitor of metalloproteinase 1  [ **TIMP1** ]  TIMP 1  [ **TIMP1** ] , and MMP  [ **MMP1** ]  3 as biologic markers of tissue damage, and also their relationship to changes in pulmonary epithelial permeability, clinical signs and symptoms, and lung structural changes.Seven serial studies were conducted in each of 8 patients undergoing chest radiotherapy RT for lung or breast cancer, beginning before the first treatment baseline and then biweekly to approximately 100 days during and after RT.  Ref: 12829155 Int J Radiat Oncol Biol Phys< MedlineTA>Int J Radiat Oncol Biol Phys, 2003
- MMP 1  [ **MMP1** ]  TIMP 1  [ **TIMP1** ]  and MMP  [ **MMP1** ]  9 TIMP 1  [ **TIMP1** ]  molar ratios were significantly increased P <.  Ref: 12824251 Invest Ophthalmol Vis Sci, 2003
- To inhibit lung MMP  [ **MMP1** ]  in rats exposed to monocrotaline 60 mg kg as a single subcutaneous injection, we used intratracheal instillation of the adenovirus mediated human TIMP 1  [ **TIMP1** ]  gene Ad.hTIMP 0 8 plaque forming units as in our previous study on hypoxic PH.  Ref: 12828857 Hum Gene Ther, 2003
- Induced sputum cells were counted and concentrations of MMP  [ **MMP1** ]  9, TIMP 1  [ **TIMP1** ]  and TNF were measured by enzyme immunoassays.  Ref: 12814147 Respir Med, 2003
- MMP  [ **MMP1** ]  3 and TIMP 1  [ **TIMP1** ]  levels are strongly positively associated with the presence of carotid lesions.  Ref: 12860260 Atherosclerosis, 2003
- Gelatinases MMP  [ **MMP1** ]  2 and MMP  [ **MMP1** ]  9, TIMP 1  [ **TIMP1** ]  expression and the extent of neovascularization in aggressive non Hodgkin s lymphomas.  Ref: 12890147 Eur J Haematol, 2003
- Tear levels of pro MMP 1  [ **MMP1** ] , pro MMP  [ **MMP1** ]  9, and TIMP 1  [ **TIMP1** ]  were measured by enzyme linked immunosorbent assay ELISA.  Ref: 12824251 Invest Ophthalmol Vis Sci, 2003
